# Human BCAS3 Expression in Embryonic Stem Cells and Vascular Precursors Suggests a Role in Human Embryogenesis and Tumor Angiogenesis

**DOI:** 10.1371/journal.pone.0001202

**Published:** 2007-11-21

**Authors:** Kavitha Siva, Parvathy Venu, Anita Mahadevan, Shankar S. K., Maneesha S. Inamdar

**Affiliations:** 1 Molecular Biology and Genetics Unit, Jawaharlal Nehru Centre for Advanced Scientific Research, Jakkur, Bangalore, India; 2 Department of Neuropathology, The National Institute of Mental Health and Neurosciences (NIMHANS), Bangalore, India; Children's Hospital Boston, United States of America

## Abstract

Cancer is often associated with multiple and progressive genetic alterations in genes that are important for normal development. BCAS3 (Breast Cancer Amplified Sequence 3) is a gene of unknown function on human chromosome 17q23, a region associated with breakpoints of several neoplasms. The normal expression pattern of BCAS3 has not been studied, though it is implicated in breast cancer progression. Rudhira, a murine WD40 domain protein that is 98% identical to BCAS3 is expressed in embryonic stem (ES) cells, erythropoiesis and angiogenesis. This suggests that BCAS3 expression also may not be restricted to mammary tissue and may have important roles in other normal as well as malignant tissues. We show that BCAS3 is also expressed in human ES cells and during their differentiation into blood vascular precursors. We find that BCAS3 is aberrantly expressed in malignant human brain lesions. In glioblastoma, hemangiopericytoma and brain abscess we note high levels of BCAS3 expression in tumor cells and some blood vessels. BCAS3 may be associated with multiple cancerous and rapidly proliferating cells and hence the expression, function and regulation of this gene merits further investigation. We suggest that BCAS3 is mis-expressed in brain tumors and could serve as a human ES cell and tumor marker.

## Introduction

Development of multicellular organisms requires controlled and co-ordinated tissue growth. Misregulation of molecular pathways that control normal embryo or tissue development can result in aberrant growth and can lead to cancer [1]. Not surprisingly, several developmentally important molecules are key mediators of the cancer phenotype when aberrantly expressed [2], [3]. Multiple and progressive genetic changes accompanied by chromosomal aberrations are often the underlying cause [4]. Human chromosome 17 has been well studied in this regard. Its complex rearrangement and duplication structure predispose it to non-allelic homologous recombinations that cause several micro-deletion disorders [5], [6]. Cruciform structures formed by direct and inverted repeats make it susceptible to one of the most common somatic rearrangement events characterized, isodicentric 17q, which is associated with several cancers and signifies poor prognosis [7]. 17q23 amplification occurs in about 20% of primary breast tumors [8]–[12]. Gain of chromosome arm 17q is the most frequent chromosomal change in neuroblastoma [13] suggesting that this region includes genes critical for tumorigenesis. An increased dosage of genes on chromosome 17q are likely to provide selective growth advantage to human ES cells for maintenance *in vitro* as human ES cells in culture show distinctive chromosomal abnormalities involving this region [14]. A comparative genomic hybridization analysis of medulloblastomas identified chromosomal imbalances with high-level amplifications involving 17q22-q24 and subsequent candidate gene analyses revealed amplification of *RPS6KB1*, *APPBP2*, *PPM1D* and *BCAS3* [15].

*BCAS3* is a large gene located at 17q23 and encodes a predicted conserved WD40 domain protein 98% identical to murine Rudhira [16]. We have shown earlier that Rudhira is a cytoplasmic protein expressed at high levels in ES cells, blood islands and the erythroid lineage during embryonic development [16]. In both the embryo and the adult Rudhira expression is seen in angiogenic precursors. The spatio-temporal expression pattern of Rudhira is well characterized but its function is not known. The developmental expression of *BCAS3*, the human ortholog of *rudhira* is not reported. However *BCAS3* is amplified in 9% of primary breast tumors [17], is expressed in tumor-derived cell lines like HeLa and MCF-7 and is implicated in breast cancer progression [16], [18]. Given this result and the observation that many aberrations of chromosome 17 map to the region of *BCAS3*, we analyzed its expression during normal development and in tumors. We used human ES cells and differentiating embryoid bodies as models of early human embryonic development and report BCAS3 expression in undifferentiated ES cells and in vascular precursors. We also show that BCAS3 is mis-expressed in different types of brain lesions that include a malignant glial tumor, a highly mitotic vascular tumor arising from meninges (hemangiopericytoma) and the newly formed channels in the wall of brain abcess. Our results suggest new roles for BCAS3 in human blood vascular development, an integral part of tumorigenesis and repair mechanism.

## Results

### Expression of BCAS3 in human ES cells and vascular precursors

**Figure 1:**
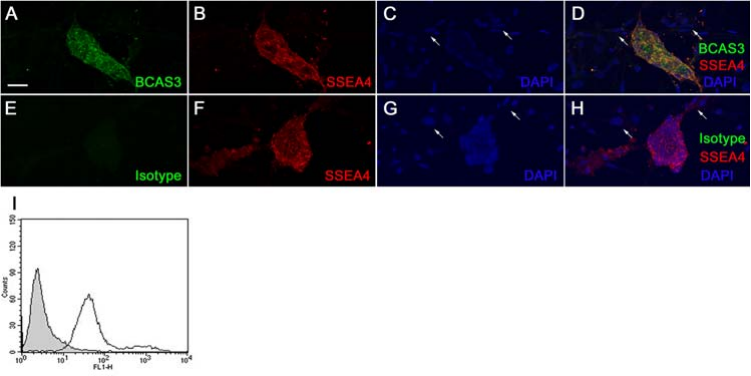
BCAS3 expression in human ES cells. Human ES cell cultures stained with (A) anti-BCAS3 antibody or (E) isotype control antibody and co-immunostained with (B, F) SSEA4. (C, G) show nuclei stained with DAPI. (D, H) are merged images of all three respective panels on their left. Arrows indicate nuclei of some feeder layer fibroblasts. (I) Flow cytometry analysis of human ES cells expressing Rudhira. Graphs show counts for Isotype control (shaded) and Rudhira positive (unshaded) cells. Scale bar: 50 µm.

As the developmental expression of human BCAS3 has not been reported due to obvious technical and ethical reasons, we began our analysis with human ES cells. Differentiation of human ES cells in culture affords a more tractable model for early human development. We found expression of BCAS3 in the cytoplasm of undifferentiated ES cells (Fig. 1 A–D). Flow cytometry analysis showed that more than 90% of undifferentiated ES cells express BCAS3 (Fig. 1I).

Having established BCAS3 expression in pluripotent cells, we examined its expression in blood vessel precursors as is observed in the case of mouse *rudhira*. Human ES cells can also spontaneously differentiate into blood islands which are precursors to embryonic blood vessels. This process is less efficient than in mouse ES cells and is often carried out under influence of growth factors. We allowed human ES cells to differentiate spontaneously into embryoid bodies (EB) that contain blood islands and nascent blood vessels. Earlier studies indicate that the maximal expression of vascular markers in human EBs is detected between week 1 and 2 of differentiation [19]. Hence we fixed EBs at 8, 10 and 14 days of differentiation and analyzed for expression of BCAS3 and cell surface markers for the blood vascular lineage.

**Figure 2:**
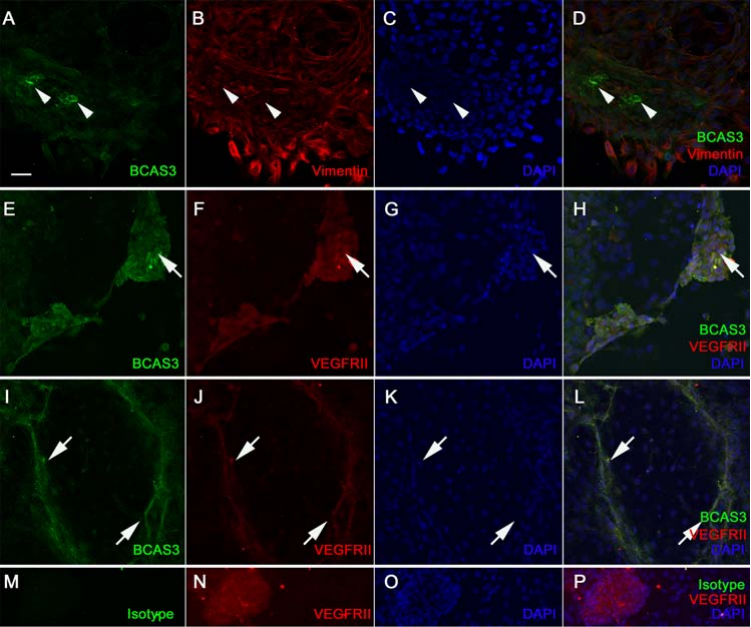
Expression of BCAS3 in embryoid bodies at day 8 of differentiation. Human EBs at day 8 of differentiation immunostained for (A, E, I) BCAS3 (green), (M) isotype control (green), (B) Vimentin (red) and (F, J, N) VEGFRII (red). (C, G, K, O) show nuclei stained with DAPI. (D, H, L, P) are merged images of respective panels to their left. Arrowheads indicate groups of cells expressing BCAS3. Arrows indicate (E–H) blood islands or (I–L) nascent vessels. Scale bar: 50 µm.

**Figure 3:**
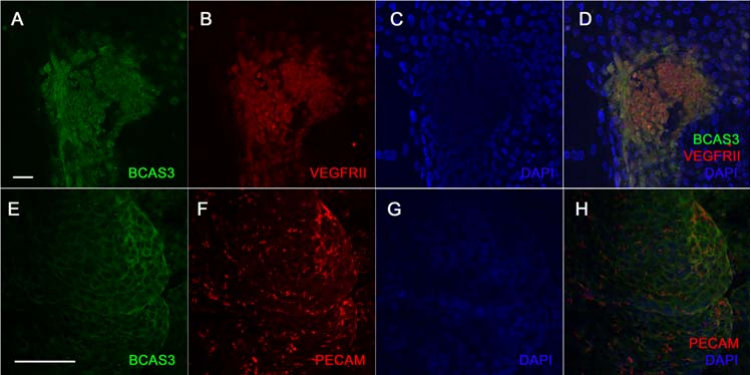
Expression of BCAS3 in embryoid bodies at day 10 of differentiation. Human EBs at day 10 of differentiation immunostained for (A, E) BCAS3 (green), (B) VEGFRII (red) and (F) PECAM (red). (C, G) show nuclei stained with DAPI. (D, H) are merged images of respective panels to their left. Scale bar: 50 µm.

**Figure 4:**
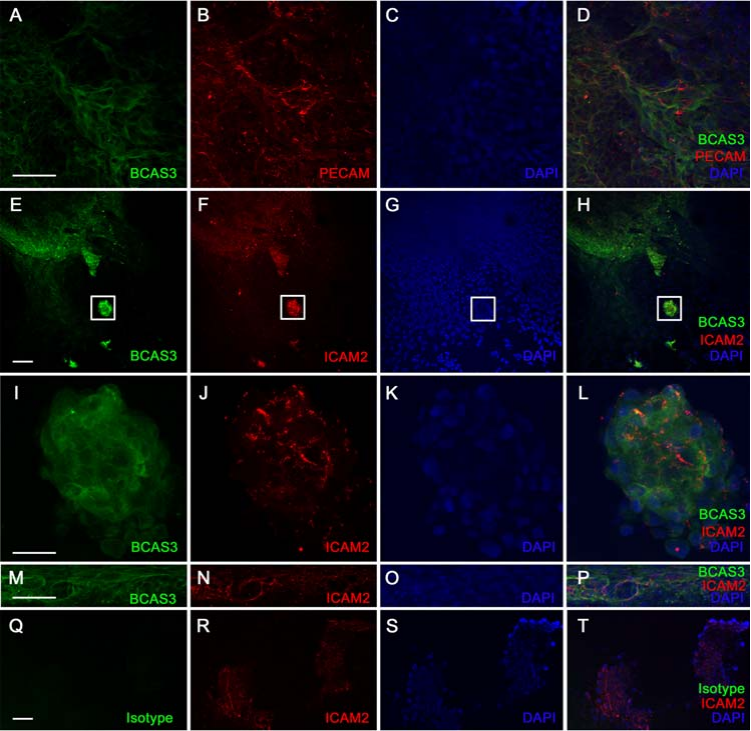
Expression of BCAS3 in embryoid bodies at day 14 of differentiation. Human EBs at day 14 of differentiation immunostained for (A, E, I, M) BCAS3 (green), (Q) isotype control (green), (B) PECAM (red) and (F, J, N, R) ICAM2 (red). (C, G, K, O, S) show nuclei stained with DAPI. (D, H, L, P, T) are merged images of respective panels to their left. Boxed area in (E–H) imaged at higher magnification shown in (I–L) respectively. Scale bar: (A, M, Q) 50 µm (E, I) 100 µm.

VEGFRII expression indicates the presence of early vascular precursors such as hemangioblasts [20]. Both VEGFRII and Platelet Endothelial Cell Adhesion Molecule (PECAM) are expressed in blood islands which have endothelial and hematopoietic precursors. Differentiated endothelial cells also express the adhesion molecule ICAM2 [21], [22]. We examined BCAS3 expression in differentiating EBS and simultaneously stained the cultures with VEGFRII or PECAM or ICAM2 to check for any co-localization with BCAS3. At day 8 of differentiation BCAS3 was detected in small clusters near the periphery of the EB. Staining for vimentin indicated that these were cells of mesodermal origin (Fig. 2 A–D). To examine whether these clusters were vascular precursors we double stained for VEGFRII. Expression of BCAS3 overlapped with VEGFRII in cell clusters resembling blood islands (Fig. 2 E–H) as well as in nascent vessels (fig. 2 I–L). Expression of BCAS3 was not uniform with some cells expressing higher BCAS3 level (Fig. 2E, H arrow). VEGFRII was also detected at day 10 in larger clusters which also expressed BCAS3 (Figure 3 A–D). BCAS3 expression was seen in day 10 EBs in clusters of cells expressing PECAM (Fig. 3 E–H). At day 14 BCAS3 expression was seen in few PECAM-expressing cells (Fig. 4 A–D) as well as in clusters of ICAM2-expressing cells (Fig. 4 E–L). On closer examination at higher magnification by confocal microscopy we found that BCAS3 and ICAM2 were not co-expressed in the same cell (Fig. 4 I–L). ICAM-2 is also a marker of differentiated endothelial cells of nascent vessels. We saw expression of BCAS3 in a few vessels that were also expressing ICAM2. However cells co-expressing BCAS3 and ICAM2 were rarely seen (Fig. 4 M–P). Immunostaining for BCAS3 with the antibody was compared to control antibody staining at all stages analyzed (Fig. 2 M–P, 3Q-T and data not shown).

### *BCAS3* mRNA but not protein is expressed in the normal brain and in glioma cell lines

**Figure 5:**
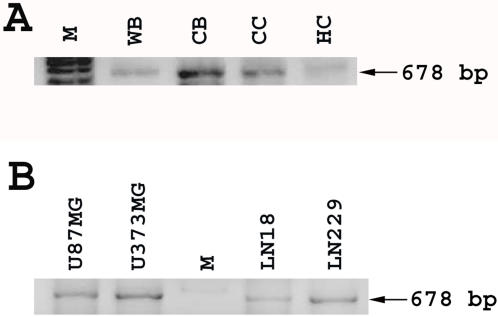
BCAS3 RNA expression in human brain and glioma cell lines. PCR analysis of cDNA from normal brain (A) and glioma cell lines (B) using BCAS3- specific primers. M: marker, WB: whole brain, CB: cerebellum, CC: Cerebral cortex, HC: hippocampus. U87MG, U373MG, LN18, LN229: glioma cell lines.

**Figure 6:**
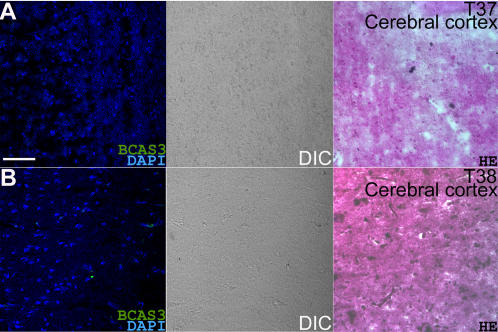
BCAS3 is not expressed in normal human frontal cerebral cortex (from two individuals). Panels to the extreme left in A and B show immunostaining on frozen sections of brain samples with anti-BCAS3 antibody, panels in the middle are DIC (differential interference contrast) images of the same sections and panels on the extreme right show adjacent sections stained with hematoxylin-eosin. Scale bar: 50 µm.

While Rudhira protein is not detected in mouse adult tissues, *rudhira* RNA can be detected in various adult tissues by RT-PCR. In particular, multiple splice variants are seen in the mouse brain [16]. To test whether this is so in human, we analyzed normal adult brain cDNA by PCR. BCAS3 amplicon was seen in cDNAs from all regions of the brain (Fig. 5A). We also amplified BCAS3 from cDNA of various glioma cell lines that represent malignant human brain tumor cells (Fig. 5B). We then examined frozen sections of human frontal cerebral cortex for BCAS3 protein expression by immunofluorescence staining. Details of samples analyzed are listed in Table 1. BCAS3 was absent from control normal (non-malignant) brain samples (Fig. 6). This is in agreement with the observation that Rudhira protein is not detected in mouse brain.

**Table 1:** Details of the samples used for the study.

Sample number	Tumor type	Grade (WHO Grade)	Age/Sex	Expression status of BCAS3
T37	Normal–Cerebral cortex	-	4/M	-
T38	Normal–Cerebral cortex	-	11/M	-
T39	Normal–Cerebral cortex	-	22/F	-
T54	Normal–Cerebral cortex	-	29/M	-
T63	Normal–Cerebral cortex	-	19/F	-
T76	Normal–Cerebral cortex	-	16/M	-
T86	Normal–Cerebral cortex	-	19/M	-
T89	Normal–Cerebral cortex	-	42/M	-
T65	Brain Abscess (organized wall)	-	67/M	-
**T66**	**Brain Abscess (vascular)**	-	**50/F**	**+**
T1350	Brain Abscess (low vascularity)	-	60/M	-
T1441	Histiocytosis (low vascularity)	-	16/M	-
T57	Atrial myxoma of heart (avascular)	-	45/M	-
T61	Atrial myxoma of heart (avascular)	-	38/F	-
T1345	Meningioma meningothelial	I	59/M	-
T62	Fibroblastic Meningioma	I	48/F	-
T67	Meningioma meningothelial	I	50/F	-
T1443	Meningioma meningothelial	I	35/F	-
T52	Atypical Meningioma	II	57/M	-
T56	Atypical Meningioma	II	48/F	-
**T1640**	**Hemangiopericytoma**	II	**20/M**	**+**
**T72**	**Hemangiopericytoma (with high mitosis)**	**III**	**35/F**	**+**
T1852	Schwannoma	I	35/M	-
T2013	Schwannoma	I	29/F	-
T1433	Paraganglioma	I	45/M	-
T1637	Plexiform neurofibroma	I	25/M	-
T77	Choroid plexus papilloma	I	22/F	-
T69	Pilocytic astrocytoma	I	33/M	-
T64	Fibrillar astrocytoma	II	6/M	-
T1628	Anaplastic astrocytoma	III	18/M	-
T70	Anaplastic astrocytoma	III	7/F	-
T59	Oligodendroglioma	II	13/M	-
T68	Oligodendroglioma	II	45/F	-
T2017	Ependyoma	III	73/F	-
T53	Oligoastrocytoma	III	48/M	-
T60	Anaplastic Oligodendroglioma	III	27/M	-
T74	Anaplastic Oligodendroglioma	III	37/F	-
**T71**	**Glioblastoma multiforme**	**IV**	**42/F**	**+**
**T1850**	**Glioblastoma multiforme**	**IV**	**45/F**	**+**
**T1866**	**Glioblastoma multiforme**	**IV**	**65/M**	**+**
**T1901**	**Glioblastoma multiforme**	**IV**	**53/F**	**+**
**T1349**	**Glioblastoma multiforme**	**IV**	**42/F**	**+**
T75	Glioblastoma (large necrotic zones)	IV	13/F	-
T73	Glioblastoma (infilterating front)	IV	45/M	-
T1439	Medulloblastoma (low vascularity)	IV	9/M	-

### BCAS3 is expressed in human brain tumors of different cell lineage and inflammatory brain abscess

**Figure 7:**
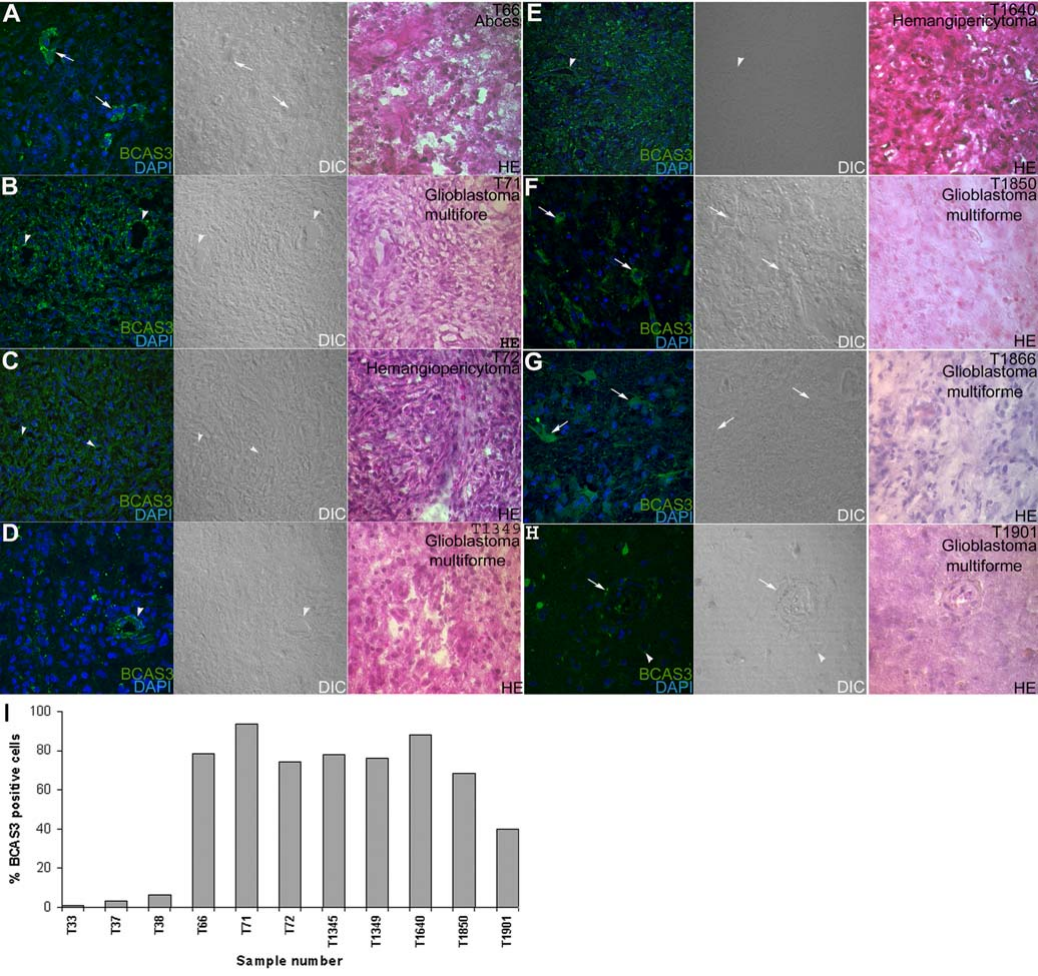
BCAS3 expression in human brain lesions. (A–H) Cryosections immunostained for BCAS3 (green). Fluorescent image with DAPI (blue) marking the nucleus or DIC image of the same field is shown as indicated. Panels to the far right show adjacent sections stained with hematoxylin-eosin. BCAS3 positive tumor cells are indicated by arrows and vessel cells by arrowheads. (I) Graph showing percent BCAS3-expressing cells in normal brain and tumor samples. Sample numbers indicated on X axis are described in Table 1.

**Figure 8:**
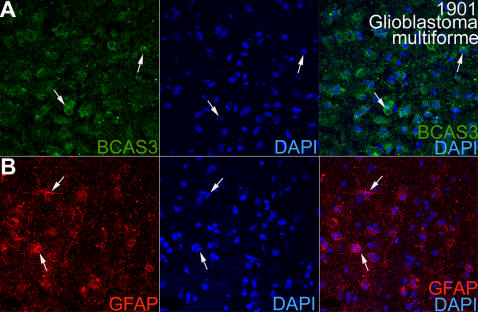
BCAS3 is expressed in glial cells in glioblastoma multiforme. Human glioblastoma sections immunostained for (A) BCAS3 (green) and (B) anti-GFAP (red). DAPI (blue) marks the nucleus. Arrows indicate glial tumor cells expressing BCAS3 (A) or GFAP (B).

Having established the expression of BCAS3 in glioma cell lines *in vitro*, we looked for similar expression in brain tumor tissue and wall of brain abscess rich in vasculature (Table 1). We found a large number of BCAS3- expressing cells in different types of brain lesions including glioblastoma, hemangiopericytoma and wall of an abscess. Eight samples showed strong immunoreactivity for BCAS3; over 50% of the cells being BCAS3^+^ (five glioblastomas, two hemangiopericytomas and one abscess) (Fig. 7I). In all the cases, BCAS3 localized to the cytoplasm (Fig. 7). Negative controls in which primary antibodies were omitted did not show any specific immunostaining. The expression of BCAS3 in the cells was rather random and showed a distinct association to vascular component of neoplasms of various cell lineages and inflammatory lesions. The labeling was noted along the endothelial aspect and the adjacent pericytes of small arterioles and endothelium of some of the larger blood vessels but not the smooth muscle cells. Labeling was seen in vascular sprouts in the wall of the brain abcess (one case) (Fig. 7A), the newly formed vessels in five cases of glioblastoma multiforme (high-grade astrocytic tumor) (Fig. 7B, D, F, G, H) and two cases of hemangiopericytoma (mesenchymal tumor of meningio-vascular origin) (Fig. 7C, E). In addition, the undifferentiated glial tumor cells in glioblastoma multiforme (grade IV) also revealed cytoplasmic labeling. The cells in most low-grade glial tumors of different cell lineages (grade I–III) failed to show expression of BCAS3 protein. BCAS3 positive cells in the tumors co-expressed GFAP (Glial Fibrillar Acidic Protein), a marker of the glial cells (Fig. 8).

## Discussion

Several cancers result from the mis-regulated expression of developmentally important genes. While there is obvious benefit in studying human tumor biology and tumor progression, elucidating gene function is often hindered by the lack of a suitable model to study the developmental and pathogenetic role of the tumor-associated genes. In this regard animal models of cancer have been extremely valuable in elucidating molecular pathways and mechanisms of disease progression. However several differences are seen in development and stem cell differentiation in humans compared to that in animal models and their cultured derivatives. Till recently, studies on developmental and pathological human gene expression have been restricted to tissues from fixed aborted fetuses, or cell- and tissue- cultures derived from them. The advent of human ES cell technology has opened up new avenues of investigation and now allows one to address developmental roles of human disease genes. We have taken advantage of this system to analyze the developmental and pathological expression of BCAS3 a gene known to be important in breast cancer. Our analysis suggests new conserved roles for BCAS3 in tumor manifestation and progression and in tissue healing.

### *BCAS3* is a conserved, developmentally expressed gene

Expression of *BCAS3* mRNA in medulloblastoma of cerebellum and BCAS3 protein in breast cancer has been reported [15], [18]. Such an expression profile suggests that BCAS3 expression is not restricted to a specific tissue. The function of BCAS3 is not known either in embryogenesis or during tumor progression. Our study suggests that *BCAS3* may have a role during embryogenesis and in tumorigenesis like many other genes that were discovered for their oncogenic role and found to be key players in embryogenesis. For example, Smad4/Dpc4, a signaling molecule of TGF-β- related pathways required for gastrulation and anterior development of the mouse embryo is involved in 50% of pancreatic cancers [23]. Similarly expression of BRCA-1, an infamous breast cancer gene, is associated with embryonic cellular proliferation and with differentiation of ectodermally- and mesodermally- derived tissues in the mouse [24], [25].

### BCAS3 is expressed in developmental and pathological angiogenesis

The kinetics of vascular marker gene expression during mouse EB differentiation is well documented and allows the detection of successive maturation steps [20]. Similar analysis done on human EBs showed that the early vascular markers VEGFRII and PECAM/CD31 appeared by the first week of differentiation [19]. The majority of early vascular markers peak between week 1 and 2 of differentiation. Hence we restricted our analysis till day 14 of differentiation. We observe that like in mouse EBs, human EBs also express BCAS3 in early stages of vascular development as indicated by VEGFRII and PECAM expression. BCAS3 expression at later stages in seen only in a few differentiated ICAM2+ endothelial cells indicating that BCAS3 expression in the vasculature is downregulated in later stages of vascular development. As seen for Rudhira in mouse EBs and mouse tissues [16] cells that co-express BCAS3 and ICAM2 are rarely detected. This suggests that the sequence of vascular gene expression in human EBs parallels that in mouse EBs. As in mouse vascular development Rudhira/BCAS3 is expressed transiently in differentiated human endothelial cells during human vascular development. This suggests an important role for BCAS3 in human angiogenesis.

BCAS3 RNA is expressed in the normal human brain and also in glial cell lines. In case of the normal brain, the cDNA represented the entire tissue including the blood vessels. Hence, the amplicon may represent RNA from the few blood vessels that express BCAS3 as seen in mouse embryos and models of angiogenesis. Rudhira is also expressed during normal physiological angiogenesis though it is not detected in differentiated endothelium. Greater the neo-vascularisation in tumors, greater is their chance of metastatic spread. As BCAS3 expression is associated with vascular precursors derived from human ES cells, it is possible that BCAS3 plays a role in the formation of tumor vessels.

We report that the protein encoded by *BCAS3* is mis-expressed in brain neo plastic and reparative lesions like glioma, abscess and hemangiopericytoma. The vessels in an abscess express enhanced levels of adhesion molecules like ICAM-1 (intercellular cell adhesion molecule) and PECAM (platelet endothelial cell adhesion molecule) which are not expressed in the normal brain and are thought to be involved in guiding the immune cells to the abscess [26]. BCAS3 is not detected in low grade tumors indicating a temporal and spatial association of BCAS3 to highly mitotic undifferentiated tumor cells and neo-angiogenesis. Similarly, many of the vascular sprouts in the wall of an abcess also showed constitutive expression, reflecting that the angiogenesis in the wall of inflammatory lesion is essentially similar to tumor blood vessels, ontologically. The vascular component- either arterioles or capillaries- in normal cerebral cortex did not show expression of BCAS3 indicating the blood vessels in normal brain are quiescent, of low turn over and the biology is distinct from vessels in tumors or active inflammatory lesions.

Blood vessels are composed of two interacting cell types. Endothelial cells form the inner lining of the vessel wall and perivascular cells referred to as pericytes, vascular smooth muscle cells (SMCs) or mural cells. Depending on their location during embryogenesis, pericytes can arise from neural crest cells or mesenchymal cells [27], [28]. Vessels associated with tumors have abnormal wall structure and branching patterns. These perivascular cells are loosely associated with the endothelium [29]. A tumor arising from these pericytes could be arising from progenitor mesenchymal cells, thus expressing BCAS3.

### BCAS3 could serve as a marker of tumor progression

BCAS3 is expressed in the neo-angiogenic component of glioblastoma, hemangiopericytoma and brain abcess wall. In addition, its expression is noted in some of the neoplastic cells of glial lineage (grade IV). Cell lines are a more homogenous population and BCAS3 expression in glioma lines is suggestive of expression in non-vascular neuroectodermal cells. This suggests that the expression of *BCAS3* RNA and constitutive presence in the tumor cells and newly formed blood vessels but not in the capillary endothelium of the normal brain is related either to the proliferative nature or an event associated with the undifferentiated and multipotent nature of the cells. The gene controlling this is probably turned off with maturation. In view of this, BCAS3 could be a surrogate marker for angiogenesis in a reparative healing process or tumor progression to higher grade of malignancy, a common phenomenon in cell biology.

*BCAS3* is located in a chromosomal region that carries several oncogenes. It is a target gene of metastasis-associated protein1 (MTA1) [18]. MTA1 is a component of the nucleosome remodeling and deacetylating (NURD) complex that contains both histone deacetylase and nucleosome remodeling activity. By expression profiling, MTA1 was identified as a target of the c-MYC oncoprotein in primary human cells and has been implicated in cellular transformation through its ability to regulate the epithelial to mesenchymal transition and metastasis [30]. MTA1 binds to estrogen response elements (ERE) on the enhancer 12 kbp downstream of the transcription start site of *BCAS3*. Breast cancer patients with high BCAS3 expression and ER/PR positive expression show impaired response to tamoxifen [31]. Hence, a study of the expression of BCAS3 can help in assessing prognosis.

We also find that most samples expressing BCAS3 are grade III and IV brain tumors though not all high-grade samples showed expression. This reflects the heterogeneity in the neoplastic cell population of brain tumors and a probable role of BCAS3 in tumor progression as seen in the breast tumors [18].

Whether expression of *BCAS3* is a cause or an effect of the general transformation and metastasis of tumors requires further studies. This is the first report on BCAS3 expression during development and in brain malignancies. The expression profile suggests that BCAS3 may play a role during both embryogenesis and tumorigenesis. An in- depth study of BCAS3 regulation and its role in progression of tumors of different organs may reveal correlation with prognosis and disease recurrence.

## Materials and Methods

### Culture and differentiation of Human ES cells

Human ES cells (HUES9) [32] (hES facility, Harvard University, Cambridge, MA, http://mcb.harvard.edu/melton/hues) were grown on irradiated mouse embryonic fibroblasts (MEFs) in medium containing 80% DMEM, 20% knockout serum, 2 mM L-glutamine, 0.1 mM β-mercaptoethanol, 1% non-essential amino acids and 4 ng/ml basic fibroblast growth factor (bFGF). For differentiation, the colonies were cut and maintained in the EB medium on bacteriological dishes without feeders. EB medium is similar to ES cell medium except the following: it is without bFGF, knockout serum is replaced by fetal calf serum, and glutamine is 4 mM. The floating EBs were allowed to attach on tissue culture dishes and fixed at different days post-attachment.

### Culture of glioma cells lines

U87MG, U373MG, LN18 and LN229 -glioma cell lines (Kind gift from M.R.S. Rao, JNCASR) were grown in DMEM containing 10% serum and 2 mM L-glutamine. Cells were scraped out of the culture dish and RNA was isolated.

### Human brain cDNAs

Marathon-Ready human brain cDNAs were obtained from Clontech (kind gift from A. Anand, JNCASR).

### Clinical material–collection and processing

Forty-six surgically resected formalin-fixed pathological tissue samples were obtained from the archives of human brain tissue repository (Human Brain Bank–a National Research facility, Department of Neuropathology, National Institute of Mental Health and Neurosciences (NIMHANS), Bangalore, India). The brains have been collected with informed consent from the close relatives after the death of the patients. This is in line with legally and ethically approved practice in India. The consent form has been approved by Institutional Scientific Ethics Committee- NIMHANS for the use of Human Biological Material for Scientific Research and Archiving.

The samples were subsequently cryopreserved in 30% sucrose solution. Cryosections 10 µm thick were picked on gelatin- coated slides for immunostaining and stored at −70°C. The age of the patients ranged between 4 years and 65 years (M∶F = 25∶21). Relatively normal control samples for comparison were cerebral cortical tissue resected during epilepsy surgery, which were free of pathology on detailed histological evaluation and were obtained with informed consent. The consent forms and the biological samples for research have been approved by Institutional Scientific Ethics Committee for the use of Human Biological Material for Scientific Research and Archiving- NIMHANS, Bangalore, India. The samples have been coded to maintain the confidentiality of the patients. The sample numbers used in the manuscript are coded and the original data is not accessible to the investigating scientist, the number indicated is used only for the purpose of this study and it cannot be tied to any other information to trace the individual. Thus the strict confidentiality is preserved as per the policy of the Human Brain Bank, NIMHANS, Bangalore. The histological diagnosis of the tissues examined is indicated in the Table 1. The brain tumors were reviewed and graded according to WHO classification for degree of malignancy.

### Reverse Transcription–Polymerase Chain Reaction (RT-PCR)

2 µg total RNA from cultured glioma cell lines was reverse transcribed using Superscript II (GIBCO) and random primers, according to the manufacturer's instructions. PCR was performed using the following BCAS3-specific primers: 466-1F 5′ GAATTCCATGGAGAGCGTCGTGACTTTTCTG 3′; 466-1R 5′ GAGCTCGAGGGACTGGTGACAGCGAATCAGC 3′

### Immunostaining and imaging

Monoclonal antibodies raised against Rudhira were used for the study [16]. As mouse Rudhira and human BCAS3 proteins are 98% identical, the anti-Rudhira antibody could be used for detecting the human protein. Antibody specificity was tested by blocking with immunogen (see Figure S1 of Text S1). Antibody was used at a dilution of 1∶10. Marker antibodies used were anti-Vimentin, anti-Flk-1, anti-PECAM and anti-ICAM2 from BD Pharmingen, GFAP (Glial fibrillar acidic protein) from Sigma Chemical Co., USA and SSEA4 from Chemicon (all at 1∶50 dilution). Alexa Fluor-conjugated secondary antibodies (Molecular Probes, USA) were used at a dilution of 1∶400 to immuno-localize the binding of the primary antibody.

Frozen sections of various brain lesions were thawed to room temperature, washed in PBS, permeabilized with 0.3% Triton X 100 and blocked for non-specific binding sites in 3% skimmed milk powder in PBS for 1 hr. Sections were incubated in monoclonal antibody (1∶10) overnight in a humid chamber. After washing off the primary antibody, the immuno-labeling of BCAS3 protein was visualized by incubating with fluorescent- labeled secondary antibody (Rabbit anti-Mouse IgG tagged to Alexa Fluor 488 1∶400) for 1 hr at RT. Sections were mounted in glycerol containing DAPI, to stain the nucleus. Adjacent sections (10 µm) were stained with hematoxylin and eosin. The fluorescent immunostained sections were viewed under a Carl-Zeiss LSM 510 Meta confocal microscope and the image analysis and processing was carried out with LSM 5 Image Browser software. Quantitation was done using MicroImage software (Roper Scientific, USA) and percentage of BCAS3-expressing (green) cells out of total number of cells (DAPI stained nuclei) in a sampled area was estimated. At least 500 cells were analyzed for each sample. Cultured cells were imaged using an Olympus IX70 fluorescence microscope and CoolSNAP-PRO*cf* camera.

### Flow cytometry analysis

Cultured cells were washed twice with PBS, trypsinized to get single cell suspension and transferred to FACS tubes. Trypsin was inactivated with 5% fetal calf serum, cells were fixed and permeabilized simultaneously with cold methanol for 1 min then washed with PBS. Non-specific binding of antibody was blocked with staining media (3% serum in PBS) for 45 min at room temperature. Staining media was then replaced with primary antibody, incubated at 4°C overnight and unbound antibody was removed with wash solution (2% serum in PBS). Cells were then incubated with fluorescent-tagged secondary antibody (Alexa Fluor 488 goat anti-mouse IgG, at 1∶400 dilution in staining media), washed and finally resuspended in 0.5 ml of PBS and run through a flow cytometer (Becton Dickinson FACS Calibur™ system). The data was analyzed using BD CellQuest Pro software.

## Supporting Information

Figure S1 Immunostaining is abolished by pre-incubation of antibody with the immunogen. (A,C,E,G) Phase contrast and (B,D,F,H) fluorescent (red) images of HEK293 cells stained with antibody preincubated with (A,B) 0 microgram (C,D) 20 microgram (E,F) 40 microgram and (G,H) 80 microgram of immunogen.
(4.96 MB TIF)

Text S1 Blocking of antibody binding 50 microliters of undiluted monoclonal anti-Rudhira/BCAS3 antibody was pre-incubated with 0, 10, 20, 40 and 80 microgram of the immunogen and at 4 oC for 12 hours, centrifuged to remove any antigen-antibody complex, diluted 1∶10 and used to stain HEK293 cells.
(0.02 MB DOC)
