# Supplementary figures and images for: Human BCAS3 Expression in Embryonic Stem Cells and Vascular Precursors Suggests a Role in Human Embryogenesis and Tumor Angiogenesis
Source: PLoS One. 2007 Nov 21;2(11):e1202. doi: 10.1371/journal.pone.0001202 (PMC2075367; doi:10.1371/journal.pone.0001202)

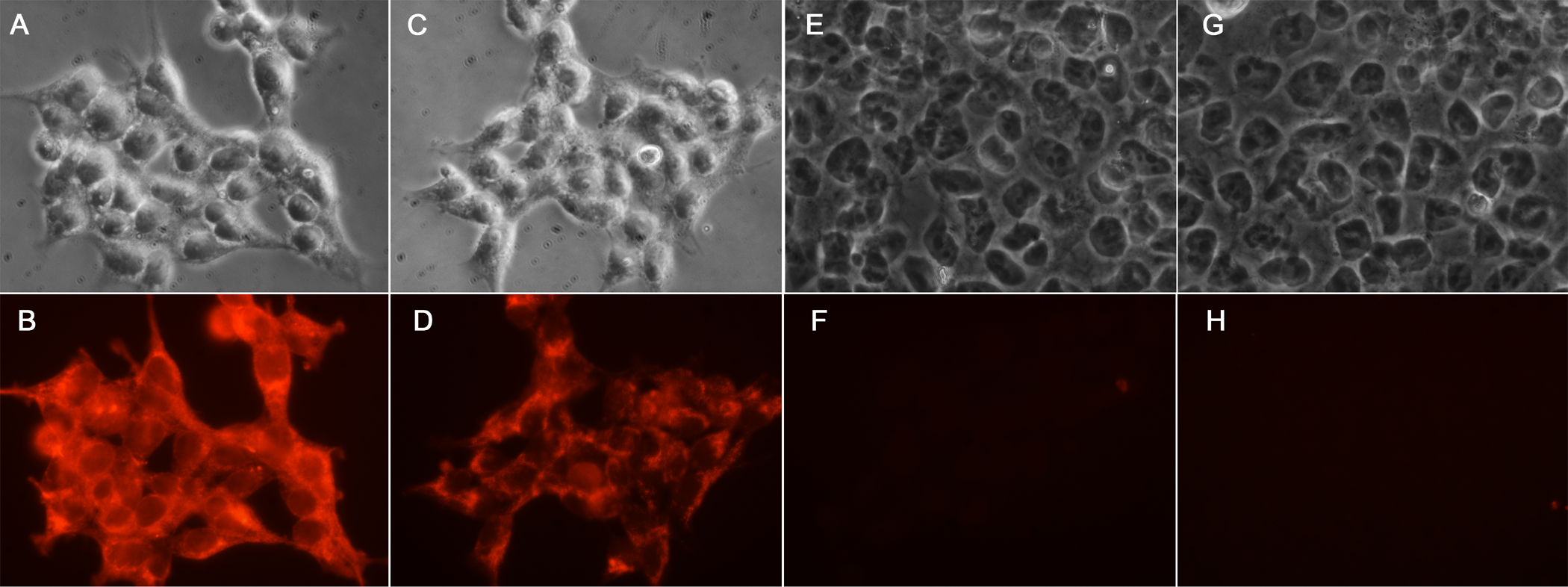

Supplement: Figure S1 — Immunostaining is abolished by pre-incubation of antibody with the immunogen. (A,C,E,G) Phase contrast and (B,D,F,H) fluorescent (red) images of HEK293 cells stained with antibody preincubated with (A,B) 0 microgram (C,D) 20 microgram (E,F) 40 microgram and (G,H) 80 microgram of immunogen. (4.96 MB TIF) [file pone.0001202.s001.tif]
