# Supplementary material for: Human BCAS3 Expression in Embryonic Stem Cells and Vascular Precursors Suggests a Role in Human Embryogenesis and Tumor Angiogenesis
Source: PLoS One. 2007 Nov 21;2(11):e1202. doi: 10.1371/journal.pone.0001202 (PMC2075367; doi:10.1371/journal.pone.0001202)
Supplement: Text S1 — Blocking of antibody binding 50 microliters of undiluted monoclonal anti-Rudhira/BCAS3 antibody was pre-incubated with 0, 10, 20, 40 and 80 microgram of the immunogen and at 4 oC for 12 hours, centrifuged to remove any antigen-antibody complex, diluted 1∶10 and used to stain HEK293 cells. (0.02 MB DOC) [file pone.0001202.s002.doc]

**Human BCAS3 Expression In Embryonic Stem Cells And Vascular Precursors Suggests A Role In Human Embryogenesis And Tumor Angiogenesis.**

**Running head: BCAS3 in development, tumors.**

**Kavitha Siva^1^, Parvathy Venu^1^, Anita Mahadevan ^2^, Shankar S K^2^ and Maneesha S. Inamdar^1^*.**

**Supplementary Data:**

**Blocking of antibody binding**

50 microliters of undiluted monoclonal anti-Rudhira/BCAS3 antibody was pre-incubated with 0, 10, 20, 40 and 80 microgram of the immunogen and at 4 ^o^C for 12 hours, centrifuged to remove any antigen-antibody complex, diluted 1:10 and used to stain HEK293 cells.
